# A social-ecological examination of physical activity and fitness among Chinese university students: a cross-sectional path analysis

**DOI:** 10.3389/fspor.2026.1775761

**Published:** 2026-05-14

**Authors:** Yong Wang, Marilou Saong

**Affiliations:** 1 Department of Public Sports, Hebei North University, Zhangjiakou City, Hebei Province, China; 2 Graduate School, University of Baguio, Baguio City, Philippines

**Keywords:** China, global health, physical activity, social-ecological model, structural equation modeling, university students

## Abstract

**Background:**

Physical inactivity and declining fitness among university students are pressing global public health concerns. While social-ecological frameworks are increasingly applied, few studies have quantitatively modeled the multilevel pathways linking institutional, familial, and behavioral factors to objectively measured fitness in non-Western contexts.

**Objective:**

This study aimed to (1) assess trends in physical fitness among Chinese university students, (2) identify key social-ecological predictors of fitness, and (3) test a path model examining direct and indirect associations consistent with ecological theory.

**Methods:**

Using a multi-source cross-sectional quantitative design, we analyzed administrative fitness records (n≈130,000) from 2019 to 2023 and a cross-sectional survey of 1,200 undergraduates in 2023. Structural equation modeling tested pathways between institutional factors, family support, physical activity (PA), and fitness.

**Results:**

Fitness scores consistently fell within the “Pass” range (60–79.9) with significant gender disparities. PA demonstrated the strongest direct association with fitness (*β*=0.38, *p* < .001). Institutional and family factors showed significant indirect effects via PA (*β*=0.18 and 0.10, respectively), while direct effects were non-significant. Lifestyle risks (screen time, sleep irregularity) negatively impacted fitness (*β*=−0.22, *p* < .001).

**Conclusion:**

Findings align with social-ecological theory, positioning PA as a key mediator between contextual factors and fitness. The study underscores the need for integrated, multilevel interventions in campus health promotion. Methodological limitations, including cross-sectional design and single-site sampling, are discussed.

## 1 Introduction

The declining physical fitness and rising sedentary behavior among university students constitute a critical global public health challenge (1, 2). Worldwide, young adults exhibit worsening cardiorespiratory capacity and increasing screen time, contributing to a concerning trend of physical inactivity (1, 3). This pattern is particularly salient in China, where national surveys have documented deteriorating health indicators among youth since the 1990s (4). In response, the *Healthy China 2030* plan was launched, prioritizing physical activity promotion, institutional reform, and health education (5, 6). Despite this ambitious policy framework, a significant proportion of Chinese university students continue to demonstrate suboptimal fitness levels, highlighting a complex interplay of individual, social, and environmental factors (7, 8).

Research has identified a broad range of determinants influencing student health, spanning subjective lifestyle factors (e.g., exercise habits, sleep patterns, motivation) and objective contextual factors (e.g., campus facilities, family support, policy environment) (9, 10). However, the extant literature is constrained by two principal limitations. First, studies often examine these variables in isolation using descriptive or bivariate analyses, which, while establishing associations, fail to elucidate the complex interactions or relative strength of pathways linking ecological determinants to health outcomes (11, 12). Second, there is a predominant focus on direct effects, with insufficient investigation into how higher-level contextual factors, such as institutional infrastructure or family environment, exert their influence indirectly by shaping health behaviors (13, 14). This gap in mechanistic, integrative modeling limits the potential to design effective, multi-level interventions (15).

The Social-Ecological Model (SE Model) offers a robust theoretical framework to address these limitations (16). It posits that health behaviors and outcomes result from dynamic interactions across multiple levels, including intrapersonal, interpersonal, institutional, and policy domains (17–19). While the SE Model has been increasingly adopted to frame qualitative explorations of student health (20, 21), quantitative studies that operationalize the full model to test complex mediating pathways remain scarce. Most existing research is confined to examining direct associations or bivariate correlations, failing to clarify how institutional or familial contexts are ultimately linked to objective fitness outcomes (12, 14).

Specifically, structural equation modeling (SEM) enables the analysis of both direct and indirect pathways across levels of association. Institutional factors, such as sports facilities or physical education curricula, may not directly determine physical fitness outcomes but may be indirectly associated with health by promoting exercise behavior and motivation. Similarly, family environments and parental support may influence health indirectly through psychosocial mechanisms such as encouragement, role modeling, and perceived competence. By operationalizing these relationships, SEM allows for the simultaneous estimation of multiple pathways and the quantification of their relative associative strengths (22–27).

This study advances the field by moving beyond the identification of isolated factors to quantitatively model the direct and indirect pathways through which multi-level factors are associated with physical fitness. We employ structural equation modeling (SEM) to test a theory-driven path model examining how institutional and family factors are associated with physical fitness indirectly, through their association with physical activity (PA). This integrative approach responds to calls for empirical research that applies social-ecological theory within a quantitative modeling framework to clarify how various layers of influence interact (15, 27).

Specifically, this study aims to:

1. Analyze longitudinal trends in the physical fitness status of university students from 2019 to 2023.
2. Identify key subjective (lifestyle) and objective (institutional, familial) factors associated with physical fitness scores.
3. Develop and test a comprehensive path model quantifying the direct and indirect associative pathways through which institutional and family environments are linked to physical fitness, primarily via PA.

## 2 Methodology

### 2.1 Study design and analytical approach

This study employed a multi-source cross-sectional quantitative design to examine associations between social-ecological factors and objectively measured physical fitness. The design integrated two distinct data sources: (1) administrative fitness records (2019–2023) for institutional trend analysis, and (2) a primary cross-sectional survey (2023) for testing a theory-driven path model.

Structural equation modeling (SEM) was used to estimate both the measurement and structural components of the model, including latent constructs and their associative pathways. Crucially, the study adheres to recent methodological guidance (28, 29) that cross-sectional data cannot establish causal mediation. Therefore, our analysis examines whether the observed pattern of direct and indirect associations is consistent with theoretical expectations, while explicitly acknowledging design limitations and alternative interpretations (e.g., reverse causality).

### 2.2 Participants and sampling

The study was conducted at Hebei North University, a large public university in northern China. Data were drawn from two independent samples.

#### 2.2.1 Administrative fitness sample (Trend analysis)

For the descriptive trend analysis, the study utilized annual fitness test records from five consecutive academic years (2019–2023), comprising approximately 130,000 records (Supplementary Files 1–S5). These data represent repeated cross-sectional measurements at the institutional level, permitting cohort-level trend tracking but not within-individual longitudinal analysis. Records were deduplicated by student ID and academic year, and cases with missing total fitness scores (<0.5%) were excluded. A detailed annual distribution of the administrative sample by grade level and gender is provided in Supplementary File 6.

#### 2.2.2 Cross-Sectional survey sample (path model)

For the primary path analysis, a new, independent sample was recruited in October 2023. The target population was all registered undergraduate students (*N* ≈ 27,000).

##### 2.2.2.1 Sample size determination

*A priori* power analysis was conducted for structural equation modeling using the semPower package in R. For a model with three latent predictors, anticipated small-to-medium effect sizes (*β* ≈ 0.20), power of 0.95, and *α* = .05, the minimum required sample size was *N* = 400. To ensure robust estimation, account for potential model complexity, and enhance generalizability within the single-institution context, the target sample size was set at *N* = 1,200.

##### 2.2.2.2 Sampling procedure

A proportionate stratified random sampling method was employed. Strata were defined by academic year (four levels: freshman to senior) and gender (two levels). Students were randomly selected from the university registry within each stratum, proportionate to the institutional population distribution.

##### 2.2.2.3 Participant flow

Of the 1,350 students invited, 1,238 provided electronic informed consent and commenced the survey (response rate = 91.7%). After data cleaning (removing incomplete responses and speeders), *N* = 1,200 valid cases were retained for analysis. The final sample maintained proportional representation across strata, as shown in Table 1.

**Table 1:** Final survey sample characteristics (*N* = 1,200).

Characteristic	n	%
Academic Year		
Freshman	312	26.0
Sophomore	288	24.0
Junior	300	25.0
Senior	300	25.0
Gender		
Male	588	49.0
Female	612	51.0

For these 1,200 participants, their objectively measured Total Physical Fitness Score from the 2023 university-administered fitness test was extracted and linked to their survey responses using a unique anonymized identifier. This linked dataset served as the basis for all inferential analyses.

### 2.3 Measures

#### 2.3.1 Outcome variable

##### 2.3.1.1 Total physical fitness score

Objectively measured using the National Student Physical Health Standards (2014 revision). The test includes seven components: height/weight (BMI), vital capacity, standing long jump, sit-and-reach, 50-meter sprint, pull-ups (males)/sit-ups (females), and 1000 m (males)/800 m (females) run. The total score is the weighted sum of standardized component scores (max = 100) plus a performance bonus (max = 20), yielding a maximum of 120 points. (See Supplementary File 7).

#### 2.3.2 Predictor variables (survey-based)

All predictors were derived from the *Student Physical Health Influencing Factors Questionnaire*, an 18-item instrument developed based on the social-ecological framework. The instrument demonstrated strong psychometric properties (see Section 2.3.3).

##### 2.3.2.1 Latent constructs (modeled in SEM)

###### 2.3.2.1.1 Physical activity (PA)

Physical Activity was modeled as a latent construct indicated by two observed variables: weekly exercise frequency and average session duration. These indicators capture complementary dimensions of engagement in physical activity behavior. A composite index (frequency  ×  duration), representing total weekly minutes of physical activity, was computed and is reported for descriptive purposes only; it was not included in the SEM to avoid redundancy and to maintain consistency with latent variable modeling principles.

###### 2.3.2.1.2 Institutional factors

A latent construct indicated by six items assessing perceived quality of sports facilities, physical education curriculum, campus sports culture, and institutional support.

###### 2.3.2.1.3 Family support

A latent construct indicated by two items: parental exercise modeling and familial encouragement for physical activity.

###### 2.3.2.1.4 Lifestyle risks

A latent construct indicated by two variables: (1) daily recreational screen time (continuous, self-reported hours per day) and (2) sleep irregularity (5-point Likert scale). These indicators were conceptualized as co-occurring behavioral risk factors reflecting a broader pattern of lifestyle dysregulation, particularly in relation to sedentary behavior and insufficient recovery, both of which are associated with adverse fitness outcomes among university students.

###### 2.3.2.1.5 Observed control variables

Gender, academic year, and body mass index (BMI) were included as covariates in the structural model.

#### 2.3.3 Instrument validity and reliability

##### 2.3.3.1 Content validity

Evaluated by ten experts in public health and exercise science. The Scale-Level Content Validity Index (S-CVI/Ave) was 0.95, exceeding the 0.80 threshold.

##### 2.3.3.2 Construct validity

Confirmatory Factor Analysis (CFA) supported the hypothesized four-factor structure (PA, Institutional, Family, Lifestyle Risks). All standardized factor loadings exceeded 0.60, with good model fit (CFI = 0.957, RMSEA = 0.045). Composite Reliability (CR) ranged from 0.81–0.88; Average Variance Extracted (AVE) ranged from 0.59–0.75, confirming convergent validity. Discriminant validity was established (√AVE > inter-construct correlations).

##### 2.3.3.3 Reliability

Internal consistency for the multi-item scales was strong: overall Cronbach’s *α* = 0.87; subscale *α* = 0.80–0.85. Test-retest reliability over two weeks (*n* = 50) was excellent (r = 0.83, *p* < .001).

The screen time item was measured as an open-ended continuous variable. While single-item continuous measures are not evaluated via internal consistency (Cronbach's alpha), they can demonstrate reliability through test-retest stability and validity through factor analytic methods (28, 29). In this study, the screen time item showed excellent test-retest reliability (r = 0.83, *p* < .001) and loaded strongly (*λ* = 0.72) on the “Lifestyle Risks” latent factor, supporting its measurement properties within the hypothesized model.

### 2.4 Data analysis

Analyses were conducted in R (v4.3) and Mplus (v8.10). The analytical strategy proceeded in three phases:

#### 2.4.1 Descriptive & trend analysis

The administrative dataset (*n* ≈ 130,000) was analyzed using ANOVAs and t-tests to characterize institutional fitness trends from 2019 to 2023.

#### 2.4.2 Measurement model

Confirmatory Factor Analysis (CFA) was conducted on the survey sample (*n* = 1,200) to validate the latent structure prior to structural modeling. Model fit was evaluated using standard criteria: CFI ≥ 0.95, TLI ≥ 0.95, RMSEA ≤ 0.06, and SRMR ≤ 0.08.

#### 2.4.3 Structural model

Structural Equation Modeling (SEM) was employed to estimate both the measurement and structural components of the hypothesized model. Institutional Factors and Family Support were specified as exogenous predictors of Physical Activity (PA), while PA and Lifestyle Risks were specified as direct predictors of Total Physical Fitness Score. Direct paths from Institutional Factors and Family Support to fitness were also estimated. Gender, academic year, and BMI were included as control variables.

To assess the potential influence of common method bias, given that institutional factors, family support, and lifestyle risks were derived from self-reported survey data, we conducted Harman's single-factor test. An unrotated exploratory factor analysis was performed on all self-reported items. The results revealed that no single factor accounted for the majority of the variance (the first factor explained 28.4% of the total variance), indicating that common method bias is unlikely to substantially influence the findings. Additionally, the confirmatory factor analysis (CFA) model with four distinct latent factors demonstrated superior fit compared to a single-factor model (*Δχ*^2^ = 487.32, *Δ*df = 6, *p* < .001), further supporting that common method bias does not pose a significant threat to the validity of the results.

Model parameters were estimated using robust maximum likelihood (MLR) to account for potential non-normality. Indirect effects were tested using bias-corrected bootstrapping (5,000 resamples). For latent constructs with two indicators (e.g., Lifestyle Risks and Family Support), model identification was achieved by fixing one factor loading to 1.0, consistent with standard SEM practice. This constraint ensures proper estimation of model parameters.

Consistent with current methodological guidance, significant indirect effects are interpreted as associative patterns consistent with mediation hypotheses, rather than evidence of causal mediation, due to the cross-sectional nature of the data (28, 29).

### 2.5 Ethical considerations

The study was approved by the University of Baguio Research Ethics Committee (See Supplementary File 8). All survey participants provided informed consent. Administrative fitness data were anonymized prior to analysis. No compensation was provided.

## 3 Results

### 3.1 Trends in physical fitness (2019–2023)

Descriptive analysis of administrative fitness records (*N* ≈ 130,000) from 2019 to 2023 revealed the physical fitness status of the student body. In accordance with the *National Student Physical Health Standards (2014 revision)*, mean total scores for all students consistently fell within the “Pass” range (60.0–79.9) across all five years (see Table 2). None of the annual or gender-specific means reached the “Good” threshold (≥80.0), indicating a moderate overall fitness level with significant room for improvement.

**Table 2:** Longitudinal trends in total physical fitness scores by gender and academic year (2019–2023).

Year	Sample Size (*N*)	Total Score, All (M ± SD)	Total Score, Male (M ± SD)	Total Score, Female (M ± SD)
2019	26,711	74.61 ± 6.72	72.52 ± 9.04	77.25 ± 6.51
2020	28,983	76.29 ± 7.98*	71.39 ± 8.86	76.21 ± 6.13
2021	27,034	74.53 ± 7.10*†	73.04 ± 8.06	76.77 ± 6.09
2022	25,925	75.07 ± 7.22*†‡	74.95 ± 7.87	78.33 ± 6.33
2023	27,318	74.84 ± 8.20*†‡§	69.30 ± 7.32	77.66 ± 5.64
Pooled	135,971	–	73.18 ± 7.47	75.77 ± 5.79*

M ± SD, mean ± standard deviation. *post-hoc* significance for yearly total scores vs. 2019.

* *p* < .05; vs. 2020.

† *p* < .05; vs. 2021.

‡ *p* < .05; vs. 2022.

§ *p* < .05. gender difference for pooled means: *p* < .01.

A one-way ANOVA indicated significant variation in mean scores across years (*p* < .01). *post-hoc* tests identified 2020 as the year with the highest mean score (76.29 ± 7.98), which was significantly higher than 2019, 2021, and 2023. The lowest mean was observed in 2021 (74.53 ± 7.10). The pattern suggests year-to-year fluctuation rather than a consistent upward or downward trend, with the 2023 mean (74.84 ± 8.20) closely resembling the 2019 baseline.

A persistent and significant gender disparity was evident (*p* < .01), with female students (pooled mea*n* = 75.77 ± 5.79) consistently outperforming male students (pooled mean = 73.18 ± 7.47) by approximately 2.6 points. This gap remained stable over time. Notably, first-year male students in the 2023 cohort scored markedly lower (69.30 ± 7.32) than their upper-year peers, while female scores remained relatively stable across academic years.

### 3.2 Key social-ecological factors (survey sample, *N* = 1,200)

Descriptive statistics for the survey sample (*N* = 1,200) are presented in Table 3. The mean objectively measured physical fitness score was 78.4 (SD = 11.8), indicating that, on average, students were within the upper range of the “Pass” classification. Participants reported an average of 124.3 min of weekly physical activity (SD = 68.0), 4.2 h of daily recreational screen time (SD = 1.8), and moderate levels of perceived institutional support and family encouragement for physical activity. These descriptive results provide an overview of the key variables before measurement and structural modeling.

**Table 3:** Descriptive statistics of Key variables (survey sample, *N* = 1,200).

Variable	Mean	SD	Min	Max	Scale/Description
Total Fitness Score	78.4	11.8	45	110	0–120 (objective)
PA: Weekly Minutes	124.3	68.0	0	480	Frequency × Duration
Screen Time (hrs/day)	4.2	1.8	1	12	Open-ended
Sleep Regularity	3.3	0.9	1	5	1 (Very Irregular) – 5 (Very Regular)
Institutional Factors	3.4	0.8	1	5	Latent construct mean
Family Support	3.2	1.1	1	5	Latent construct mean
BMI (kg/m2)	21.8	3.2	15.1	32.6	Continuous

### 3.3 Measurement model evaluation

Before testing the structural relationships, a Confirmatory Factor Analysis (CFA) was conducted to evaluate the measurement model comprising four latent constructs: Physical Activity (PA), Institutional Factors, Family Support, and Lifestyle Risks. The measurement model demonstrated good fit to the data (CFI = 0.957, RMSEA = 0.045), supporting the adequacy of the hypothesized factor structure. All standardized factor loadings exceeded 0.60, indicating strong indicator reliability. Composite reliability (CR) values ranged from 0.81 to 0.88, and average variance extracted (AVE) values ranged from 0.59 to 0.75, confirming convergent validity. Discriminant validity was also established, as the square root of AVE for each construct exceeded inter-construct correlations.

For latent constructs with two indicators (Physical Activity, Family Support, and Lifestyle Risks), model identification was achieved by fixing one factor loading to 1.0, consistent with standard SEM practice. Overall, these results support the adequacy of the measurement model for subsequent structural analysis.

### 3.4 Structural equation modeling results

The hypothesized Structural Equation Model (SEM) demonstrated excellent fit to the data (*χ*^2^/df = 1.52, CFI = 0.968, TLI = 0.960, RMSEA = 0.039 [90% CI: 0.033–0.045], SRMR = 0.042) and explained 44% of the variance in total physical fitness scores. As shown in Table 4, Physical Activity (PA) exhibited the strongest positive direct association with physical fitness (*β* = 0.38, *p* < .001), indicating that higher engagement in physical activity is associated with better fitness outcomes. In contrast, Lifestyle Risks (screen time and sleep irregularity) showed a significant negative direct association with fitness (*β* = –0.22, *p* < .001), suggesting that greater lifestyle risk is linked to lower fitness levels. Direct effects of Institutional Factors (*β* = 0.07, *p* = .212) and Family Support (*β* = 0.04, *p* = .401) on fitness were not statistically significant. Among the control variables, gender (*β* = –0.08, *p* = .014) and BMI (*β* = –0.11, *p* = .003) were significant predictors, whereas academic year was not (*p* = .351).

**Table 4:** Standardized direct, indirect, and total effects from the structural equation model (*N* = 1,200).

Predictor	Direct Effect (*β* [95% CI])	Indirect Effect via PA (*β* [95% CI])	Total Effect (*β*)
PA	0.38*** [0.30, 0.46]	–	0.38***
Institutional Factors	0.07 [–0.04, 0.18]	0.18** [0.08, 0.28]	0.25**
Family Support	0.04 [–0.05, 0.13]	0.10* [0.03, 0.17]	0.14*
Lifestyle Risks	–0.22*** [–0.31, –0.13]	–	–0.22***

CI, Bias-corrected bootstrap confidence interval (5,000 samples).

Consistent with our analytical approach, significant indirect paths are interpreted as associative patterns consistent with theoretical mediation hypotheses. The cross-sectional design precludes causal inference regarding mediation.

* *p* < .05.

** *p* < .01.

*** *p* < .001.

Further analysis of indirect effects revealed that both Institutional Factors and Family Support were significantly associated with physical fitness indirectly through Physical Activity. Specifically, Institutional Factors demonstrated a significant indirect effect via PA (*β* = 0.18, 95% CI [0.08, 0.28], *p* < .01), with a corresponding total effect of *β* = 0.25 (*p* < .01). Similarly, Family Support showed a significant indirect effect through PA (*β* = 0.10, 95% CI [0.03, 0.17], *p* < .05), with a total effect of *β* = 0.14 (*p* < .05). These findings indicate that higher-level contextual factors are associated with fitness outcomes primarily through their relationship with physical activity, rather than through direct effects.

## 4 Discussion

This study employed an SE Model to examine the multilevel factors associated with physical fitness among Chinese university students. Integrating institutional trend data with a large-scale cross-sectional survey, we identified key patterns consistent with ecological theory. The results underscore that student fitness is not solely an individual behavioral outcome but is significantly shaped by institutional and familial contexts, primarily through their influence on physical activity (PA).

### 4.1 Fitness trends and disparities

Consistent with national reports, the longitudinal data (2019–2023) revealed that the average fitness of students consistently met the minimum “Pass” standard but failed to reach the “Good” benchmark (4, 5). This suggests a persistent population-level fitness deficit within Chinese higher education, aligning with global concerns regarding declining physical capacity in young adults (1, 2).

The observed fluctuation in fitness scores, with a peak in 2020 followed by a decline in subsequent years, aligns with emerging empirical evidence on the impact of the COVID-19 pandemic on student fitness in China. While the temporary increase in 2020 may reflect short-term behavioral adaptations or institutional responses, multiple longitudinal studies indicate that this pattern was not sustained. For instance, analyses of Chinese university cohorts have documented short-term stability or improvement in certain fitness indicators during the early pandemic period, followed by significant declines in endurance performance and increases in overweight and obesity by 2021–2022 (30–33). Similarly, provincial and multi-university datasets show rising failure rates and deteriorating physical fitness indicators in the years following COVID-19 disruptions, particularly in cardiorespiratory endurance. These findings suggest that the 2020 peak observed in the present study may represent a transitional effect, rather than a sustained improvement, reflecting the complex and time-dependent impact of pandemic-related restrictions on student behavior and physical activity patterns. This interpretation is further supported by international evidence demonstrating reductions in physical activity and fitness during lockdown periods, with incomplete recovery thereafter (34, 35).

The stable and significant gender gap, with female students outperforming males, corroborates earlier findings in Chinese contexts (5, 36). This disparity may be attributed to several factors, including higher obesity prevalence and lower cardio-respiratory performance among male students, as well as potentially differing social norms and motivations for physical activity (36, 37). The notably low scores among first-year males highlight a critical window for intervention during the transition to university life, a period often marked by reduced structure and increased behavioral autonomy (10, 23).

### 4.2 Central role of physical activity and lifestyle risks

The path analysis confirmed PA as the strongest direct correlate of physical fitness(*β* = 0.38), reinforcing its undisputed role as a cornerstone of health (1, 2). This finding emphasizes that interventions aiming to improve fitness must prioritize increasing the frequency and regularity of PA, not just its duration or intensity. Concurrently, Lifestyle Risks, operationalized as high screen time and irregular sleep, exerted a significant direct negative effect (*β* = –0.22). This aligns with evidence linking sedentary behavior and poor sleep hygiene to reduced metabolic health, impaired recovery, and lower fitness (2, 38–40). The dual prominence of PA and lifestyle risks underscores a behavioral syndemic where promoting healthy behaviors and mitigating risky ones are equally critical.

### 4.3 Social-ecological pathways: the mediating role of institutional and family contexts

The most novel contribution of this study lies in quantifying the indirect pathway through which higher-order ecological factors relate to fitness. Contrary to models assuming direct environmental effects, our results show that Institutional Factors (e.g., facility quality, supportive curriculum) and Family Support were not directly associated with fitness scores. Instead, their influence was fully mediated through PA.

This pattern strongly supports the core proposition of social-ecological theory: environmental and social factors shape health by enabling or constraining health-promoting behaviors (16, 17). A supportive campus environment does not automatically make students fitter; rather, it increases the likelihood that they will engage in regular PA, which then improves fitness (25, 26). This pattern suggests that the primary value of institutional factors (including facilities and curriculum) is not in directly causing better fitness, but in creating an environment that facilitates and motivates the voluntary physical activity, which, in turn, improves health. Simply having access to facilities and a well-designed curriculum may be insufficient if students lack the motivation, time, or social cues to use them actively. This illustrates that institutional environments primarily foster health by providing the motivation and opportunity for regular activity (25, 26, 41).

Similarly, family support, through modeling and encouragement, fosters positive exercise identities and habits that persist into young adulthood (24, 42). The significant indirect effects (*β* = 0.18 and *β* = 0.10 for institutional and family factors, respectively) quantify this enabling role, providing empirical support for investing in these contextual domains as a strategy to promote PA and, consequently, fitness.

### 4.4 Implications for policy and practice

The identified pathways suggest that effective campus health promotion must be multi-level and integrated**.**

1. Strengthening Institutional Enablers: Universities should move beyond merely providing facilities. Investment should focus on creating accessible, appealing, and inclusive physical environments (e.g., extended hours, diverse activity options, intramural leagues) and integrating PA promotion into academic policy and campus culture (6, 36, 43).
2. Engaging Families as Partners: Given the enduring influence of family, universities can develop communication strategies to engage parents in fostering active lifestyles, even during the university years, through shared information and family-inclusive events.
3. Targeting Behavioral Synergies: Programs should concurrently promote PA and mitigate lifestyle risks. This could involve integrating “active breaks” into academic schedules, implementing screen-time awareness campaigns linked to sleep hygiene, and offering time-management workshops that prioritize physical wellness (37, 38).
4. Tailoring Interventions for At-Risk Groups: The significant gender and year-level disparities call for targeted outreach, particularly for first-year male students, through orientation programs, peer mentorship, and tailored incentive structures.

These strategies align with the goals of *Healthy China 2030* and offer a blueprint for ecologically informed student health promotion with potential applicability in global higher education settings (5, 26).

### 4.5 Limitations and future directions

The findings of this study should be interpreted in light of several limitations. First, the cross-sectional design of the survey data precludes causal inference. Although the structural equation modeling (SEM) results are consistent with theoretically derived pathways, the directionality of associations cannot be definitively established, and alternative explanations, including reverse causality, remain plausible. Future research employing longitudinal or experimental designs is necessary to establish temporal ordering and causal mechanisms.

Second, the use of a single institutional sample from Hebei North University limits the external validity of the findings. The university is situated in a northern region characterized by prolonged cold winters, which may restrict outdoor physical activity for extended periods. Although institutional provisions for sports and fitness exist, access to indoor facilities and their utilization may not fully compensate for seasonal constraints. Moreover, the relatively homogeneous regional student population may reflect shared socio-cultural orientations toward physical activity and academic priorities. These contextual conditions differ from those of universities in other parts of China, particularly southern and coastal regions with milder climates and more diverse infrastructure. As such, the observed associations may be context-specific, limiting generalizability to other institutional and geographic settings.

Third, several key variables, particularly physical activity behaviors, screen time, and sleep patterns, were assessed using self-reported measures, which are subject to recall bias and social desirability effects. Although efforts were made to ensure reliability and validity, future studies should incorporate objective measures (e.g., accelerometry, digital usage tracking, or wearable devices) to enhance measurement precision.

Finally, while this study operationalized a multi-level framework based on the social-ecological model, the analysis remained limited to quantitative indicators. The absence of qualitative data restricts a deeper understanding of the contextual and experiential mechanisms through which institutional and family factors influence physical activity behaviors.

Future research should address these limitations by: (1) employing longitudinal or cross-lagged panel designs to clarify causal pathways; (2) expanding to multi-institutional and cross-regional samples to enhance generalizability; (3) integrating objective behavioral measures to complement self-reports; and (4) incorporating qualitative approaches to capture students’ lived experiences and contextual influences on physical activity. Such efforts will strengthen the empirical basis for developing ecologically grounded and context-sensitive interventions in university health promotion.

## 5 Conclusion

This study demonstrates that the physical fitness of Chinese university students is a product of interconnected social-ecological systems. While PA is the proximal behavioral driver of fitness, its adoption is significantly influenced by perceived institutional support and family encouragement. Conversely, lifestyle risks like excessive screen time pose a direct threat to fitness. These results argue against siloed health promotion efforts and instead advocate for comprehensive, ecologically grounded strategies that simultaneously enhance supportive environments, foster healthy behaviors, and mitigate risk factors. By adopting such an integrated approach, universities can more effectively contribute to the national *Healthy China 2030* ambition and address the global challenge of declining fitness among young adults.

## Data Availability

The original contributions presented in the study are included in the article/Supplementary Material, further inquiries can be directed to the corresponding author.
