# Supplementary material for: A social-ecological examination of physical activity and fitness among Chinese university students: a cross-sectional path analysis
Source: Front Sports Act Living. 2026 May 14;8:1775761. doi: 10.3389/fspor.2026.1775761 (PMC13216034; doi:10.3389/fspor.2026.1775761)
Supplement: Supplementary file 6 [file Table6.docx]

**The Table shows the distribution of students by grade level and gender for each year.** In this context, a student's "class" (e.g., Class of 2019) refers to their **year of entry as freshmen.** Therefore, the data can be interpreted as follows:

**Cross-Sectional (Vertical) Reading:** Each column shows the composition of the entire student body in that academic year. For example, in **2021**, the student body was composed of: The **Class of 2021** as freshmen (n=7,263); The **Class of 2020** as sophomores (n=6,530); The **Class of 2019** as juniors (n=6,848); The **Class of 2018** as seniors (n=6,393)

**Cohort (Diagonal) Reading:** Each entering class (cohort) can be tracked across its four years. For instance, the **Class of 2019** can be traced as: Freshmen in **2019**(n=7,222); Sophomores in **2020**(n=7,222); Juniors in **2021** (n=6,848); Seniors in **2022**(n=5,366)

The changing cohort numbers from year to year reflect normal student attrition and retention. This dataset was used exclusively for the **descriptive trend analysis in Section 3.1,** which provides an objective, institutional-level context. **Because the data were not linked at the individual level across years, these analyses reflect aggregate cohort-level trends, not within-individual change.**

Table. Distribution of Hebei students according to grade level and gender (2019-2023)

| **Year, Grade** | **2019** | **2020** | **2021** | **2022** | **2023** |
| --- | --- | --- | --- | --- | --- |
| College freshman | 7222 | 7016 | 7263 | 7236 | 7161 |
| Sophomore | 6724 | 7222 | 6530 | 6844 | 6899 |
| Third year in university | 6830 | 7368 | 6848 | 6389 | 6700 |
| Senior year | 5935 | 7377 | 6393 | 5366 | 6558 |
| **Total** | **26711** | **28983** | **27034** | **25925** | **27318** |
| **Grade** | 7222 | 7016 | 7263 | 7236 | 7161 |
| **Male** | **59550** | | | | |
| **Female** | **76421** | | | | |
