# Supplementary material for: A social-ecological examination of physical activity and fitness among Chinese university students: a cross-sectional path analysis
Source: Front Sports Act Living. 2026 May 14;8:1775761. doi: 10.3389/fspor.2026.1775761 (PMC13216034; doi:10.3389/fspor.2026.1775761)
Supplement: Supplementary file 7 [file Table7.docx]

**Data scoring criteria**

National Physical Health Standards for Students (revised in 2014)

*BMI Individual rating scale (unit: kg/m^2^)*

| **Lv.** | **Single score** | **Schoolboy** | **Schoolgirl** |
| --- | --- | --- | --- |
| **Normal** | 100 | 17.9~23.9 | 17.2~23.9 |
| **Underweight** | 80 | ≤17.8 | ≤17.1 |
| **Overweight** |  | 24.0~27.9 | 24.0~27.9 |
| **Obesity** | 60 | ≥28.0 | ≥28.0 |

**Descriptions for the Physical Fitness Level**

**Outstanding** – very much exceeded the Chinese National Student Physical Fitness Standard; demonstrates a very high level of performance, endurance, and strength in executing the tasks or activities

**Good** – exceeded the Chinese National Student Physical Fitness Standard; demonstrates an above average performance, endurance, and strength in executing the tasks or activities

**Pass** - within the Chinese National Student Physical Fitness Standard; demonstrates an average performance, endurance, and strength in executing the tasks or activities

**Fail** – below the Chinese National Student Physical Fitness Standard; demonstrates a below average performance, endurance, and strength in executing the tasks or activities

*Individual rating table of vital capacity (unit: ml)*

| **Lv.** | **Single score** | **Schoolboy** | | **Schoolgirl** | |
| --- | --- | --- | --- | --- | --- |
|  |  | **Freshman year**  **Sophomore year** | **Junior year**  **Senior year** | **Freshman year**  **Sophomore year** | **Junior year**  **Senior year** |
| **Outstanding** | **100** | 3400 | 3450 | 5040 | 5140 |
|  | **95** | 3350 | 3400 | 4920 | 5020 |
|  | **90** | 3300 | 3350 | 4800 | 4900 |
| **Good** | **85** | 3150 | 3200 | 4550 | 4650 |
|  | **80** | 3000 | 3050 | 4300 | 4400 |
| **Pass** | **78** | 2900 | 2950 | 4180 | 4280 |
|  | **76** | 2800 | 2850 | 4060 | 4160 |
|  | **74** | 2700 | 2750 | 3940 | 4040 |
|  | **72** | 2600 | 2650 | 3820 | 3920 |
|  | **70** | 2500 | 2550 | 3700 | 3800 |
|  | **68** | 2400 | 2450 | 3580 | 3680 |
|  | **66** | 2300 | 2350 | 3460 | 3560 |
|  | **64** | 2200 | 2250 | 3340 | 3440 |
|  | **62** | 2100 | 2150 | 3220 | 3320 |
|  | **60** | 2000 | 2050 | 3100 | 3200 |
| **Flunk** | **50** | 1960 | 2010 | 2940 | 3030 |
|  | **40** | 1920 | 1970 | 2780 | 2860 |
|  | **30** | 1880 | 1930 | 2620 | 2690 |
|  | **20** | 1840 | 1890 | 2460 | 2520 |
|  | **10** | 1800 | 1850 | 2300 | 2350 |

*Individual score Table for 50m race (unit: seconds)*

| **Lv.** | **Single score** | **Schoolboy** | | **Schoolgirl** | |
| --- | --- | --- | --- | --- | --- |
|  |  | **Freshman year**  **Sophomore year** | **Junior year**  **Senior year** | **Freshman year**  **Sophomore year** | **Junior year**  **Senior year** |
| **Outstanding** | **100** | 7.5 | 7.4 | 6.7 | 6.6 |
|  | **95** | 7.6 | 7.5 | 6.8 | 6.7 |
|  | **90** | 7.7 | 7.6 | 6.9 | 6.8 |
| **Good** | **85** | 8.0 | 7.9 | 7.0 | 6.9 |
|  | **80** | 8.3 | 8.2 | 7.1 | 7.0 |
| **Pass** | **78** | 8.5 | 8.4 | 7.3 | 7.2 |
|  | **76** | 8.7 | 8.6 | 7.5 | 7.4 |
|  | **74** | 8.9 | 8.8 | 7.7 | 7.6 |
|  | **72** | 9.1 | 9.0 | 7.9 | 7.8 |
|  | **70** | 9.3 | 9.2 | 8.1 | 8.0 |
|  | **68** | 9.5 | 9.4 | 8.3 | 8.2 |
|  | **66** | 9.7 | 9.6 | 8.5 | 8.4 |
|  | **64** | 9.9 | 9.8 | 8.7 | 8.6 |
|  | **62** | 10.1 | 10.0 | 8.9 | 8.8 |
|  | **60** | 10.3 | 10.2 | 9.1 | 9.0 |
| **Flunk** | **50** | 10.5 | 10.4 | 9.3 | 9.2 |
|  | **40** | 10.7 | 10.6 | 9.5 | 9.4 |
|  | **30** | 10.9 | 10.8 | 9.7 | 9.6 |
|  | **20** | 11.1 | 11.0 | 9.9 | 9.8 |
|  | **10** | 11.3 | 11.2 | 10.1 | 10.0 |

*Single score table for seated forward flexion (unit: cm)*

| **Lv.** | **Single score** | **Schoolboy** | | **Schoolgirl** | |
| --- | --- | --- | --- | --- | --- |
|  |  | **Freshman year**  **Sophomore year** | **Junior year**  **Senior year** | **Freshman year**  **Sophomore year** | **Junior year**  **Senior year** |
| **Outstanding** | **100** | 25.8 | 26.3 | 24.9 | 25.1 |
|  | **95** | 24.0 | 24.4 | 23.1 | 23.3 |
|  | **90** | 22.2 | 22.4 | 21.3 | 21.5 |
| **Good** | **85** | 20.6 | 21.0 | 19.5 | 19.9 |
|  | **80** | 19.0 | 19.5 | 17.7 | 18.2 |
| **Pass** | **78** | 17.7 | 18.2 | 16.3 | 16.8 |
|  | **76** | 16.4 | 16.9 | 14.9 | 15.4 |
|  | **74** | 15.1 | 15.6 | 13.5 | 14.0 |
|  | **72** | 13.8 | 14.3 | 12.1 | 12.6 |
|  | **70** | 12.5 | 13.0 | 10.7 | 11.2 |
|  | **68** | 11.2 | 11.7 | 9.3 | 9.8 |
|  | **66** | 9.9 | 10.4 | 7.9 | 8.4 |
|  | **64** | 8.6 | 9.1 | 6.5 | 7.0 |
|  | **62** | 7.3 | 7.8 | 5.1 | 5.6 |
|  | **60** | 6.0 | 6.5 | 3.7 | 4.2 |
| **Flunk** | **50** | 5.2 | 5.7 | 2.7 | 3.2 |
|  | **40** | 4.4 | 4.9 | 1.7 | 2.2 |
|  | **30** | 3.6 | 4.1 | 0.7 | 1.2 |
|  | **20** | 2.8 | 3.3 | -0.3 | 0.2 |
|  | **10** | 2.0 | 2.5 | -1.3 | -0.8 |

*Individual Score Table of Standing Long jump (unit: cm)*

| **Lv.** | **Single score** | **Schoolboy** | | **Schoolgirl** | |
| --- | --- | --- | --- | --- | --- |
|  |  | **Freshman year**  **Sophomore year** | **Junior year**  **Senior year** | **Freshman year**  **Sophomore year** | **Junior year**  **Senior year** |
| **Outstanding** | **100** | 207 | 208 | 273 | 275 |
|  | **95** | 201 | 202 | 268 | 270 |
|  | **90** | 195 | 196 | 263 | 265 |
| **Good** | **85** | 188 | 189 | 256 | 258 |
|  | **80** | 181 | 182 | 248 | 250 |
| **Pass** | **78** | 178 | 179 | 244 | 246 |
|  | **76** | 175 | 176 | 240 | 242 |
|  | **74** | 172 | 173 | 236 | 238 |
|  | **72** | 169 | 170 | 232 | 234 |
|  | **70** | 166 | 167 | 228 | 230 |
|  | **68** | 163 | 164 | 224 | 226 |
|  | **66** | 160 | 161 | 220 | 222 |
|  | **64** | 157 | 158 | 216 | 218 |
|  | **62** | 154 | 155 | 212 | 214 |
|  | **60** | 151 | 152 | 208 | 210 |
| **Flunk** | **50** | 146 | 147 | 203 | 205 |
|  | **40** | 141 | 142 | 198 | 200 |
|  | **30** | 136 | 137 | 193 | 195 |
|  | **20** | 131 | 132 | 188 | 190 |
|  | **10** | 126 | 127 | 183 | 185 |

*One-minute sit-ups and one-minute pull-ups (unit: time)*

| **Lv.** | **Single score** | **schoolboy** | | **schoolgirl** | |
| --- | --- | --- | --- | --- | --- |
|  |  | **Freshman year**  **Sophomore year** | **Junior year**  **Senior year** | **Freshman year**  **Sophomore year** | **Junior year**  **Senior year** |
| **Outstanding** | **100** | 56 | 57 | 19 | 20 |
|  | **95** | 54 | 55 | 18 | 19 |
|  | **90** | 52 | 53 | 17 | 18 |
| **Good** | **85** | 49 | 50 | 16 | 17 |
|  | **80** | 46 | 47 | 15 | 16 |
| **Pass** | **78** | 44 | 45 |  |  |
|  | **76** | 42 | 43 | 14 | 15 |
|  | **74** | 40 | 41 |  |  |
|  | **72** | 38 | 39 | 13 | 14 |
|  | **70** | 36 | 37 |  |  |
|  | **68** | 34 | 35 | 12 | 13 |
|  | **66** | 32 | 33 |  |  |
|  | **64** | 30 | 31 | 11 | 12 |
|  | **62** | 28 | 29 |  |  |
|  | **60** | 26 | 27 | 10 | 11 |
| **Flunk** | **50** | 24 | 25 | 9 | 10 |
|  | **40** | 22 | 23 | 8 | 9 |
|  | **30** | 20 | 21 | 7 | 8 |
|  | **20** | 18 | 19 | 6 | 7 |
|  | **10** | 16 | 17 | 5 | 6 |

Note: Boys: pull-ups; Girl: Sit-ups.
